# The when and how of the gynaecological examination: a survey among Norwegian general practitioners

**DOI:** 10.1080/02813432.2019.1619829

**Published:** 2019-05-29

**Authors:** Stefán Hjörleifsson, Bjørn Bjorvatn, Eivind Meland, Guri Rørtveit, Yngvild Hannestad, Gunnar Tschudi Bondevik

**Affiliations:** a Department of Global Public Health and Primary Care, University of Bergen, Bergen, Norway;; b Research Unit for General Practice, NORCE Norwegian Research Centre, Bergen, Norway;; c National Centre for Emergency Primary Health Care, Uni Health Research, Bergen, Norway

**Keywords:** Gynaecological examination, practice variation, bimanual palpation, physician gender

## Abstract

**Introduction:** Little is known about the indications general practitioners (GPs) perceive as relevant for performing gynaecological examinations (GEs), how GPs master the GE and associated procedures, and how they handle the sensitive nature of GEs.

**Methods:** In 2015, 70 medical students at the University of Bergen distributed a questionnaire to all 175 GPs in the practices they visited. The questions covered practical routines related to GEs, insertion of intrauterine device, frequency of GEs in different clinical settings and use of assisting personnel. Statistical analyses included chi-square tests and multiple logistic regressions adjusting for age, gender, specialization and localization.

**Results:** Ninety male and 61 female GPs (87% of invited GPs) responded to the questionnaire. A minority (8%) usually had other staff present during GEs. Compared with female colleagues, male GPs performed bimanual palpation significantly less often in connection with routine Pap smear (AOR 0.3 (95% CI 0.1-0.6)). Twenty-eight percent of the GPs stated that they often/always omitted the GE if the patient was anxious about GE and 35% when the patient asked for referral to a gynaecologist. Omission was more frequent among male GPs. When the GP decided to refer to a gynaecologist based on the patient’s symptoms, more male than female GPs omitted GE (AOR 2.5 (95% CI 1.1-5.4)).

**Conclusion:** Male gender of the GP may be associated with barriers to medical evaluation of pelvic symptoms in women, potentially leading to substandard care. Possibly, however, male GPs’ reluctance to perform the GE may also limit unnecessary bimanual palpation in asymptomatic women.

## Introduction

The gynaecological examination (GE) is a procedure that women may experience as embarrassing, painful and even threatening, and there is limited guidance on decision making, and technical as well as interpersonal aspects of the GE [1]. While many women have a positive attitude to the medical benefits of GEs [2], they sometimes have adverse expectations when preparing for the procedure, and may experience the procedure negatively [3]. This calls for interpersonal sensitivity and rigorous professional judgement on the doctor’s side in determining when and how to perform a GE. Professional bodies in many countries promote the use of chaperones to manage the ethically sensitive nature of GEs.

In Norway, general practitioners (GPs) are supposed to provide comprehensive medical care for their patients and a national registered list patient system provides each inhabitant with a designated GP. Normally, women need referral from the GP to visit a gynaecologist, although commercial services are available in some cities. GPs and primary care midwifes provide maternity care to most pregnant women, and GPs insert intrauterine devices (IUDs) and take Pap smears. These arrangements are in accordance with the primary health care ideal of comprehensiveness which has been championed in Norway and is promoted by the WHO as a core feature of good health care organisation [4]. However, it is known that GPs' delivery of services sometimes is subject to undue practice variation, and concerns have been raised that the comprehensiveness and thus clinical quality of primary health care may be threatened [4,5].

No official guidelines exist in Norway on the clinical, technical and interpersonal aspects of GE in primary care. A publication by the Norwegian Medical Association on practical procedures for postgraduate GP training gives clear advice on all of these, but this publication is not mandatory curriculum for GPs in Norway [6]. And to our knowledge no research has been done to ascertain the indications GPs perceive as relevant for performing GEs. Neither is it known how GPs manage technical aspects of this procedure, or how they manage and negotiate the invasive nature of the GE. We therefore decided to perform this study, aiming to investigate how Norwegian GPs manage technical and interpersonal aspects of the GE and what factors they take into account when considering whether to perform this procedure.

## Material and methods

During practical training in general practice in January–March 2015, 70 final year medical students at the University of Bergen, Norway, distributed a questionnaire to all 175 GPs in the practices they visited. The one-page questionnaire comprised questions about routines related to GEs, including the presence of other staff during GEs, whether bimanual palpation was done when women came for Pap smears, GE in antenatal and postnatal care, and whether the GPs inserted IUDs. It also included questions about circumstances where the GPs decided not to do a GE although the patient presented with a gynaecological problem. The completed questionnaires were anonymous.

The GPs responded using a five-point scale: ‘never’, ‘seldom’, ‘sometimes’, ‘often’, and ‘always’. We recorded the GPs' age, gender, specialization in family medicine, and localization of practice (urban vs. rural). Multiple logistic regression was used for the binary dependent variable of answering ‘often’ or ‘always’ vs. ‘never’, ‘seldom’ or ‘sometimes’. The odds ratio for a defined category of an independent variable approximates the adjusted risk, relative to its reference category, of answering ‘often’ or ‘always’. We performed statistical analyses using IBM SPSS Statistics version 24, and included univariate and multiple logistic regression with unadjusted (OR) and adjusted odds ratios (AOR), and 95% confidence intervals (CI), and we set the level of statistical significance to .05. The AORs are based on multiple logistic regression analyses adjusted for the potential confounders included in the tables; GPs' age, gender, specialization and localization of practice.

We conducted the study in compliance with the ethical guidelines of the Helsinki Declaration. All participants received written information about the purpose of the study, and this included assurance that we would collect the data anonymously. The study was approved by the Norwegian Social Science Data Services – the governmental agency for protecting survey research respondents’ privacy according to the Norwegian Personal Data Act (Ref. No. 2015/41460). As the study did not include patients and was not affected by the Norwegian Health Research Act, approval from an ethics committee was not required. Each GP practice received two medical textbooks to compensate for their participation.

## Results

We received responses from 152 GPs (87% of the invited). Of these, 40% were female (the Norwegian national average is 42% [7]), 57% were certified specialists in general practice (national average 53%), 43% were younger than 40 years, and 76% younger than 55 years (national figures 33% and 67%, respectively), and 41% worked in rural practices.

We found large variations in GE routines. Nearly 90% of the GPs never or seldom had other staff present during GEs (Table 1). However, a significantly higher proportion of male GPs would often or always have other staff present, AOR 9.11 (95% CI 1.11–74.85), as compared to female GPs. Eleven per cent of male GPs always had other staff present, whereas 42% never did. When the patient came solely for a Pap smear, male GPs did bimanual palpation significantly less frequently than female GPs, AOR 0.40 (95% CI 0.20–0.80).

**Table 1.** Routines among 152 GPs when performing GE – Is a chaperone present and is bimanual palpation performed when patients consult for Pap smear only?

		*n*	Never	Seldom	Sometimes	Often	Always	OR Often/Always	AOR Often/Always
			*n* (%)	*n* (%)	*n* (%)	*n* (%)	*n* (%)	(95%CI)	(95%CI)
Chaperone^a^									
Total		152	79 (52)	54 (36)	7 (5)	1 (1)	11 (7)		
GP gender	Female	61	41 (67)	18 (30)	1 (2)	0	1 (2)	1.00	1.00
	Male	90	38 (42)	35 (39)	6 (7)	1 (1)	10 (11)	**8.35 (1.05–66.50)**	**9.11 (1.11–74.85)**
GP age	≤34	38	17 (45)	17 (45)	1 (3)	0	3 (8)	1.00	1.00
(years)	35–49	66	38 (58)	22 (33)	1 (2)	0	5 (8)	0.96 (0.22–4.25)	1.46 (0.24–8.82)
	≥50	48	24 (50)	15 (31)	5 (10)	1 (2)	3 (6)	1.06 (0.22–5.05)	1.23 (0.17–9.13)
Location	Urban	85	48 (57)	26 (31)	4 (5)	1 (1)	6 (7)	1.00	1.00
	Rural	62	26 (42)	28 (45)	3 (5)	0	5 (8)	0.98 (0.30–3.24)	1.06 (0.30–3.81)
GP certified	Yes	87	48 (55)	27 (31)	5 (6)	1 (1)	6 (7)	1.00	1.00
	No	53	26 (49)	20 (38)	2 (4)	0	5 (9)	1.19 (0.35–3.96)	1.83 (0.38–8.92)
Bimanual palpation^b^									
Total		149	13 (9)	22 (15)	31 (21)	30 (20)	53 (36)		
GP gender	Female	60	3 (5)	2 (3)	14 (23)	12 (20)	29 (48)	1.00	1.00
	Male	88	10 (11)	20 (23)	17 (19)	17 (19)	24 (27)	**0.40 (0.20–0.80)**	**0.29 (0.13–0.64)**
GP age	≤34	37	4 (11)	7 (19)	6 (16)	7 (19)	13 (35)	1.00	1.00
(years)	35–49	65	6 (9)	8 (12)	19 (29)	12 (19)	20 (31)	0.82 (0.37–1.85)	0.86 (0.29–2.57)
	≥50	47	3 (6)	7 (15)	6 (13)	11 (23)	20 (43)	1.65 (0.68–3.99)	2.29 (0.67–7.84)
Location	Urban	83	5 (6)	14 (17)	16 (19)	17 (21)	31 (37)	1.00	1.00
	Rural	61	7 (12)	7 (12)	12 (20)	13 (21)	22 (36)	0.98 (0.50–1.92)	0.78 (0.37–1.64)
GP certified	Yes	85	8 (9)	10 (12)	17 (20)	20 (24)	30 (35)	1.00	1.00
	No	52	4 (8)	10 (19)	10 (19)	10 (19)	18 (35)	0.82 (0.40–1.64)	0.87 (0.32–2.33)

OR (odds ratio) and adjusted odds ratio (AOR) with 95%CI (confidence interval) for answering ‘Often’ or ‘Always’ vs. ‘Never’, ‘Seldom’ or ‘Sometimes’ based on univariate and multiple logistic regression adjusted for the variables included in the table. Results in bold types are statistically significant at .05 level.

a Question: ‘Is a chaperone present when you perform GE?’.

b Question: ‘Do you perform bimanual palpation when a woman consults for Pap smear only?’.

Missing responses among 152 study participants: 0–15.

Nearly three quarters of the GPs never or seldom performed a GE when pregnant women came for their first antenatal check-up (Table 2). However, a significantly higher proportion of GPs practicing in rural areas would often or always do GE in this situation, AOR 5.72 (95% CI 1.01–32.54). Approximately one third of the GPs never or seldom did a GE when women came for their postnatal check-up. Nearly half of the female GPs would always or often do GE in postnatal women, their male colleagues did this significantly less often, AOR 0.24 (95% CI 0.10–0.56). A significantly higher proportion of GPs ≥ 50 years performed GE in women coming for postnatal check-ups compared to GPs 34 years or younger, AOR 13.09 (95% CI 2.92–58.80). Nearly one fifth of the GPs did not insert IUDs (Table 3).

**Table 2.** Routines among 152 GPs when performing GE – Is GE included at the first antenatal and postnatal consultation?

		*n*	Never	Seldom	Sometimes	Often	Always	OR Often/always	AOR Often/always
			*n* (%)	*n* (%)	*n* (%)	*n* (%)	*n* (%)	(95%CI)	(95%CI)
GE at first antenatal consultation^a^									
Total		150	44 (29)	66 (44)	28 (19)	11 (7)	1 (1)		
GP gender	Female	61	10 (16)	33 (54)	15 (25)	2 (3)	1 (2)	1.00	1.00
	Male	88	34 (39)	33 (38)	13 (15)	8 (9)	0	1.93 (0.49–7.60)	1.33 (0.21–8.42)
GP age	≤34	38	13 (34)	18 (47)	6 (16)	1 (3)	0	1.00	1.00
(years)	35–49	66	22 (33)	31 (47)	13 (20)	0	0	NA	NA
	≥50	46	9 (20)	17 (37)	9 (20)	10 (22)	1 (2)	**11.63 (1.43–94.83)**	NA
Location	Urban	83	25 (30)	39 (47)	16 (19)	3 (4)	0	1.00	1.00
	Rural	62	16 (26)	27 (44)	10 (16)	8 (13)	1 (2)	**4.53 (1.17–17.50)**	**5.72 (1.01–32.54)**
GP certified	Yes	85	25 (29)	36 (42)	14 (17)	9 (11)	1 (1)	1.00	1.00
	No	53	14 (26)	27 (51)	11 (21)	1 (2)	0	0.14 (0.18–1.16)	0.52 (0.05–6.03)
GE post partum^b^									
Total		149	16 (11)	39 (26)	46 (31)	34 (23)	14 (9)		
GP gender	Female	61	0	16 (26)	17 (28)	19 (31)	9 (15)	1.00	1.00
	Male	87	16 (18)	23 (26)	29 (33)	14 (16)	5 (6)	**0.33 (0.16–0.67)**	**0.24 (0.10–0.56)**
GP age	≤34	38	8 (21)	13 (34)	9 (24)	6 (16)	2 (5)	1.00	1.00
(years)	35–49	65	5 (8)	15 (23)	28 (43)	12 (19)	5 (8)	1.33 (0.51–3.46)	2.97 (0.80–11.08)
	≥50	46	3 (7)	11 (24)	9 (20)	16 (35)	7 (15)	**3.75 (1.42–9.90)**	**13.09 (2.92–58.80)**
Location	Urban	83	9 (11)	20 (24)	28 (34)	18 (22)	8 (10)	1.00	1.00
	Rural	61	5 (8)	18 (30)	16 (26)	16 (26)	6 (10)	1.24 (0.62–2.49)	0.95 (0.43–2.12)
GP certified	Yes	84	7 (8)	21 (25)	26 (31)	20 (24)	10 (12)	1.00	1.00
	No	53	8 (15)	15 (28)	15 (28)	11 (21)	4 (8)	0.71 (0.33–1.50)	1.79 (0.60–5.34)

OR (odds ratio) and adjusted odds ratio (AOR) with 95%CI (confidence interval) for answering ‘Often’ or ‘Always’ vs. ‘Never’, ‘Seldom’ or ‘Sometimes’ based on univariate and multiple logistic regression adjusted for the variables included in the table. Results in bold types are statistically significant at .05 level.

a Question: ‘Do you perform GE at the first antenatal visit?’.

b Question: ‘Do you perform GE at the postnatal visit?’.

NA = Not applicable due to insufficient items in cell.

Missing responses among 152 study participants: 2–15.

**Table 3.** Do GPs (*n* = 152) insert IUDs?

		*n*	Yes *n* (%)	OR Yes (95%CI)	AOR Yes (95%CI)
Total		151	125 (83)		
GP gender	Female	61	54 (88)	1.00	1.00
	Male	89	70 (79)	0.48 (0.19–1.22)	0.39 (0.13–1.17)
GP age	≤34	38	24 (63)	1.00	1.00
(years)	35–49	65	59 (91)	**5.74 (1.97–16.68)**	2.78 (0.72–10.74)
	≥50	48	42 (88)	**4.08 (1.39–12.02)**	1.51 (0.33–7.01)
Location	Urban	84	64 (76)	1.00	1.00
	Rural	62	56 (90)	**2.92 (1.09–7.77)**	2.20 (0.76–6.38)
GP certified	Yes	87	79 (91)	1.00	1.00
	No	52	37 (71)	**0.25 (0.10–0.64)**	0.31 (0.08–1.19)

OR (odds ratio) and adjusted odds ratio (AOR) with 95%CI (confidence interval) for answering ‘Yes’ based on univariate and multiple logistic regression adjusted for the variables included in the table. Results in bold types are statistically significant at .05 level.

Missing responses among 152 study participants: 1–16.

If the patients presenting a gynaecological problem showed anxiety or shyness, approximately one quarter of the GPs would often or always refrain from doing a GE (Table 4). Male GPs would significantly more often than female GPs refrain from doing a GE in these circumstances, AOR 3.75 (95% CI 1.50–9.40). Similarly, if perceiving the relationship between the GP and the patient as an obstacle, male GPs would significantly more often refrain from performing a GE, AOR 2.43 (95% CI 1.12–5.25).

**Table 4.** Do GPs (*n* = 152) refrain from GE if the patient shows anxiety or embarrassment, or due to their relationship with the patient?

		*n*	Never	Seldom	Sometimes	Often	Always	OR Often/always	AOR Often/always
			*n* (%)	*n* (%)	*n* (%)	*n* (%)	*n* (%)	(95%CI)	(95%CI)
Anxiety/Embarrassment^a^									
Total		150	13 (9)	47 (31)	48 (32)	32 (21)	10 (7)		
GP gender	Female	61	9 (15)	28 (46)	16 (26)	6 (10)	2 (3)	1.00	1.00
	Male	88	4 (5)	19 (22)	31 (35)	26 (30)	8 (9)	**4.17 (1.77–9.84)**	**3.75 (1.50–9.40)**
GP age	≤34	38	3 (8)	11 (29)	12 (32)	11 (29)	1 (3)	1.00	1.00
(years)	35–49	65	5 (8)	20 (31)	24 (37)	12 (19)	4 (6)	0.71 (0.29–1.72)	1.25 (0.38–4.12)
	≥50	47	5 (11)	16 (34)	12 (26)	9 (19)	5 (11)	0.92 (0.36–2.32)	1.48 (0.40–5.53)
Location	Urban	84	9 (11)	24 (29)	22 (26)	23 (27)	6 (7)	1.00	1.00
	Rural	61	2 (3)	22 (36)	24 (41)	8 (13)	4 (7)	0.46 (0.21–1.01)	0.53 (0.23–1.21)
Specialist	Yes	86	6 (7)	29 (34)	30 (35)	17 (20)	4 (5)	1.00	1.00
	No	52	5 (10)	14 (27)	16 (31)	12 (23)	5 (10)	1.50 (0.70–3.22)	2.17 (0.74–6.39)
Relationship^b^									
Total		144	18 (13)	32 (22)	33 (23)	36 (25)	25 (17)		
GP gender	Female	56	12 (21)	19 (34)	10 (18)	12 (21)	3 (5)	1.00	1.00
	Male	87	6 (7)	13 (15)	23 (26)	23 (26)	22 (25)	**2.93 (1.42–6.05)**	**2.43 (1.12–5.25)**
GP age	≤34	33	1 (3)	4 (12)	12 (36)	11 (33)	5 (15)	1.00	1.00
(years)	35–49	65	11 (17)	14 (22)	16 (25)	14 (22)	10 (15)	0.62 (0.27–1.45)	0.78 (0.26–2.32)
	≥50	46	6 (13)	14 (30)	5 (11)	11 (24)	10 (22)	0.89 (0.36–2.19)	0.86 (0.26–2.89)
Location	Urban	81	10 (12)	13 (16)	21 (26)	21 (26)	16 (20)	1.00	1.00
	Rural	58	5 (9)	19 (33)	11 (19)	14 (24)	9 (16)	0.78 (0.39–1.55)	0.80 (0.39–1.66)
GP certified	Yes	84	10 (12)	24 (29)	15 (18)	22 (26)	13 (16)	1.00	1.00
	No	48	7 (15)	5 (10)	14 (29)	12 (25)	10 (21)	1.19 (0.58–2.42)	1.25 (0.48–3.30)

OR (odds ratio) and adjusted odds ratio (AOR) with 95%CI (confidence interval) for answering ‘Often’ or ‘Always’ vs. ‘Never’, ‘Seldom’ or ‘Sometimes’ based on univariate and multiple logistic regression adjusted for the variables included in the table. Results in bold types are statistically significant at .05 level.

a Question: ‘When a patient consults for a gynaecological problem, do you omit the GE if the patient expresses anxiety or embarrassment for this procedure?’.

b Question: ‘When a patient consults for a gynaecological problem, do you omit the GE if your relationship with the patient makes this procedure difficult to perfom?’.

Missing responses among 152 study participants: 2–20.

One third of the GPs would often or always omit doing a GE if the woman requested referral to a gynaecologist (Table 5). Male GPs significantly more often than female GPs refrained from doing a GE in these circumstances, AOR 3.10 (95% CI 1.34–7.20). If the patient presented a gynaecological problem that the GP judged to be in need of referral to a specialist, 37% of the GPs would always or often refrain from doing a GE. In this situation, male GPs significantly more often refrained from doing a GE compared to their female colleagues, AOR 2.48 (95% CI 1.14–5.40).

**Table 5.** Do GPs (*n* = 152) refrain from GE if the patient requests referral to a gynaecologist or if she presents a gynaecological problem that the GP judges to be in need of referral to a specialist?

		*n*	Never	Seldom	Sometimes	Often	Always	OR Often/Always	AOR Often/Always
			*n* (%)	*n* (%)	*n* (%)	*n* (%)	*n* (%)	(95%CI)	(95%CI)
Patient request^a^									
Total		150	14 (9)	39 (26)	45 (30)	37 (25)	15 (10)		
GP gender	Female	60	11 (18)	20 (33)	18 (30)	8 (13)	3 (5)	1.00	1.00
	Male	89	3 (3)	19 (21)	26 (29)	29 (33)	12 (14)	**3.81 (1.75–8.26)**	**3.10 (1.34–7.20)**
GP age	≤34	38	4 (11)	8 (21)	14 (37)	10 (26)	2 (5)	1.00	1.00
(years)	35–49	66	9 (14)	21 (32)	19 (29)	13 (20)	4 (6)	0.75 (0.31–1.81)	0.62 (0.19–2.05)
	≥50	46	1 (2)	10 (22)	12 (26)	14 (30)	9 (20)	2.17 (0.89–5.31)	1.68 (0.48–5.96)
Location	Urban	84	9 (11)	23 (27)	22 (26)	21 (25)	9 (11)	1.00	1.00
	Rural	61	4 (7)	16 (26)	20 (33)	15 (25)	6 (10)	0.95 (0.47–1.89)	1.08 (0.50–2.33)
GP certified	Yes	85	4 (5)	22 (26)	27 (32)	23 (27)	9 (11)	1.00	1.00
	No	53	8 (15)	15 (28)	15 (28)	11 (21)	4 (8)	0.65 (0.31–1.37)	0.86 (0.30–2.48)
Gynaecological problem^b^									
Total		151	15 (10)	39 (26)	41 (27)	39 (26)	17 (11)		
GP gender	Female	61	9 (15)	22 (36)	16 (26)	11 (18)	3 (5)	1.00	1.00
	Male	89	6 (7)	17 (19)	24 (27)	28 (32)	14 (16)	**3.00 (1.45–6.21)**	**2.48 (1.14–5.40)**
GP age	≤34	38	4 (11)	10 (26)	12 (32)	11 (29)	1 (3)	1.00	1.00
(years)	35–49	66	8 (12)	19 (29)	17 (26)	15 (23)	7 (11)	1.08 (0.46–2.55)	0.82 (0.27–2.51)
	≥50	47	3 (6)	10 (21)	12 (26)	13 (28)	9 (19)	1.91 (0.78–4.65)	1.39 (0.41–4.73)
Location	Urban	85	8 (9)	18 (21)	27 (32)	22 (26)	10 (12)	1.00	1.00
	Rural	61	6 (10)	20 (33)	14 (23)	15 (25)	6 (10)	0.87 (0.44–1.73)	0.86 (0.42–1.80)
GP certified	Yes	86	5 (6)	24 (28)	23 (27)	22 (26)	12 (14)	1.00	1.00
	No	53	8 (15)	13 (25)	14 (26)	15 (28)	3 (6)	0.79 (0.38–1.61)	0.88 (0.32–2.39)

OR (odds ratio) and adjusted odds ratio (AOR) with 95%CI (confidence interval) for answering ‘Often’ or ‘Always’ vs. ‘Never’, ‘Seldom’ or ‘Sometimes’ based on univariate and multiple logistic regression adjusted for the variables included in the table. Results in bold types are statistically significant at .05 level.

a Question: ‘When a patient consults for a gynaecological problem, do you omit the GE if the patient asks for referral to a gynaecologist for her problem?’.

b Question: ‘When a patient consults for a gynaecological problem, do you omit the GE if you have decided to refer the patient to a gynaecologist in any case?’.

Missing responses among 152 study participants: 2–14.

## Discussion

This study indicates large practice variation in routines and use of GEs, especially according to the GP’s gender. Compared to female GPs, male GPs more often had other staff present during GEs, did bimanual palpation less often when patients came for Pap smears, and more often omitted performing a GE when the patient presented with a gynaecological problem.

GPs have a professional duty to manage and minimise the intrusive and embarrassing impact of the GE, while also ensuring that the procedure is performed when required. However, even with the best intentions and professional conduct, some patients may still find the GE so stressful that the GP has to omit the procedure – and it is not unlikely that this happens more often with male GPs. It is also known that male medical students can be uncomfortable with performing the GE [8], and our findings indicate that some male GPs may carry this discomfort with them and thus try to avoid the procedure. While sensitivity to gendered ethical issues is a virtue, GPs should not use their own discomfort or the potentially disturbing nature of GEs as an excuse for providing sub-standard clinical care for their patients. Avoiding GE for non-medical reasons may put women with gynaecological problems at risk of delayed diagnosis, under-treatment or unnecessary worries.

Even in situations when the GP promptly refers a patient with symptoms to a gynaecologist, the omission of GE can potentially be harmful since lack of information will make it more difficult for the gynaecologist to know which priority to give to the patient. On the other hand, there may be occasions when it is acceptable to avoid repeated GEs. Our study did not provide sufficient information to determine to what extent the GPs’ omission of a GE was acceptable from a clinical perspective.

Although long-acting reversible contraceptives are safe and effective and IUDs are important to women’s sexual health [9], nearly one fifth of the GPs in our study did not insert IUDs. This is in accordance with Pahle et al’s registry study; only three out of four GPs in Norway were reimbursed in 2013 for insertion of IUDs [10]. The same study found considerable practice variation in IUD-insertions depending on the GPs gender, with female GPs having higher odds for performing IUD-insertions (OR 6.28, 95% CI 4.70–8.82) than male GPs. Our study did not detect the same gender related practice variation in IUD insertion.

Half of the GPs in this study reported performing bimanual palpation always or often when consulted for Pap smear only. As there is no evidence of clinical benefit of screening asymptomatic women with bimanual palpation, and this procedure carries costs and risks for harm [11] that include false positive findings and overtreatment [12], this may be regarded as medical over-activity. The Norwegian Medical Association’s textbook for practical procedures for GP specialist candidates highlights that one potential justification for performing bimanual palpation without expecting significant findings is if this helps clinicians improve or maintain their practical skills [6]. On the other hand, the textbook also urges GPs to exercise critical judgement as to whether and how extensively they need to submit each patient to a GE. Extrapolating benefit beyond evidence has been implicated as a common mechanism of medical overuse, i.e. procedures that are useful in a particular setting are performed also in a different setting where not useful [13]. This can lead to overdiagnosis and false positive findings, increase health concerns and healthcare expenditure, and lead to underuse by directing resources away from more important tasks [14]. In a 2012 survey of US physicians, 68% of gynaecologists, and 39% of general practitioners reported that they routinely performed GE for cancer screening [15]. Our findings may indicate that Norwegian GPs do not fully discriminate between clinical situations where there is an indication for bimanual palpation and routine check-ups, and situations where this may be omitted.

We found that male GPs did bimanual palpation less frequently than female GPs when consulted for a Pap smear. The same pattern was found for first antenatal appointment and in postnatal care. The omission of palpation in the routine ante- and postnatal situation is in accordance with Norwegian national guidelines [16] and textbooks [17]. No previous research explains this difference between male and female GPs. Male GPs’ possible insecurity with GEs and their sensitivity to patients’ distress may be one reason why they are less prone to perform an examination which may not be beneficial for the patient. Conversely, female GPs may be more risk-aversive in this context than their male colleagues in the sense that they fear missing a diagnosis, as has been shown in some other clinical situations [18], and thus be more prone to perform bimanual palpation. Furthermore, the finding that a lower proportion of GPs less than 50 years included GE in antenatal and postnatal care as compared to older colleagues, may indicate that younger GPs have a higher uptake of recent evidence based recommendations.

A survey of 1000 women visiting a family-planning clinic in the UK found that overall, one-third of the women actively did not want a chaperone. On the other hand, 11% preferred a chaperone to be present when undergoing GE by a female doctor, and 62% preferred a chaperone when the doctor was male [19]. In a Canadian survey of 350 women visiting their general practitioner, the corresponding figures for preferring a chaperone during pelvic and breast examinations were 62% for male GPs and 30% for female GPs [20].

A possible reason why provision of a professional chaperone may be difficult in general practice is lack of available staff. A survey among GPs in Canada found that the availability of a nurse was associated with use of chaperones [21]. On the other hand, in Norway, personal continuity of care and trust between patient and doctor is considered to be a cornerstone of primary care, and national surveys consistently indicate high levels of trust in GPs [22]. This may lead GPs to conclude – correctly or not – that a chaperone is not needed. The argument can even be made that chaperones sometimes interfere with doctor-patient rapport and erode trust. The fact remains that surveys from other countries consistently demonstrate that some women want a chaperone to be present during GE, and that more patients prefer a chaperone if the doctor is male [23]. Although we found that a higher proportion of male GPs had other staff present during GE as compared to female GPs, the confidence interval for this finding was broad and the overall proportion of GPs who reported using chaperones was quite low.

While the high response rate (87%) in this study was a strength, the answers given may not represent a fully correct picture of Norwegian GPs’ practice. Our informants, being GPs taking on medical students for practical training, may not be representative of all GPs in Norway, and their age distribution was somewhat lower than that of all GPs in Norway. On the other hand, the similarity of our findings on insertion of IUDs (83% of GPs) with those in Pahle et al’s registry study (75% of GPs), seems to support the internal and external validity of our questionnaire approach.

## Conclusion

Male gender of the GP may be associated with barriers to medical evaluation of pelvic symptoms in women, potentially leading to substandard care. However, male GPs’ refraining from bimanual palpation when asymptomatic women consult for a Pap smear may also limit the use of an examination of questionable utility.
